# Arylamine *N*-acetyltransferase 1 deficiency inhibits drug-induced cell death in breast cancer cells: switch from cytochrome C-dependent apoptosis to necroptosis

**DOI:** 10.1007/s10549-022-06668-3

**Published:** 2022-08-02

**Authors:** Courtney E. McAleese, Neville J. Butcher, Rodney F. Minchin

**Affiliations:** School of Biomedical Sciences, The University of Queensland, Brisbane, QLD 4072 Australia

**Keywords:** NAT1, Breast cancer, Apoptosis, Drug resistance, Necroptosis

## Abstract

**Purpose:**

Arylamine *N*-acetyltransferase 1 (NAT1) deficiency has been associated with drug resistance and poor outcomes in breast cancer patients. The current study aimed to investigate drug resistance in vitro using normal breast cancer cell lines and NAT1-deficient cell lines to understand the changes induced by the lack of NAT1 that resulted in poor drug response.

**Methods:**

The response to seven chemotherapeutic agents was quantified following NAT1 deletion using CRISPR-Cas 9 in MDA-MB-231 and T-47D cells. Apoptosis was monitored by annexin V staining and caspase 3/7 activity. Cytochrome C release and caspase 8 and 9 activities were measured by Western blots. Caspase 8 was inhibited using Z-IETD-FMK and necroptosis was inhibited using necrostatin and necrosulfonamide.

**Results:**

Compared to parental cells, NAT1 depleted cells were resistant to drug treatment. This could be reversed following NAT1 rescue of the NAT1 deleted cells. Release of cytochrome C in response to treatment was decreased in the NAT1 depleted cells, suggesting suppression of the intrinsic apoptotic pathway. In addition, NAT1 knockout resulted in a decrease in caspase 8 activation. Treatment with necrosulfonamide showed that NAT1 deficient cells switched from intrinsic apoptosis to necroptosis when treated with the anti-cancer drug cisplatin.

**Conclusions:**

NAT1 deficiency can switch cell death from apoptosis to necroptosis resulting in decreased response to cytotoxic drugs. The absence of NAT1 in patient tumours may be a useful biomarker for selecting alternative treatments in a subset of breast cancer patients.

**Supplementary Information:**

The online version contains supplementary material available at 10.1007/s10549-022-06668-3.

## Introduction

Molecular characterisation of breast cancer subtypes has led to the discovery of novel therapeutics that have changed how the disease is treated. For example, patients with oestrogen receptor positive tumours are usually offered endocrine therapy in the first instance while those with HER2 receptor positive tumours benefit from targeted therapies such as trastuzumab and lapatinib. Patients with tumours classified as triple negative are primarily treated with combination chemotherapy because targeted therapies for this group have been slow to emerge [1]. Regardless of the tumour subtype, a large proportion of patients either do not respond, or develop resistance to their treatment [2, 3]. Accordingly, better markers that can predict and monitor drug resistance are urgently needed.

Arylamine *N*-acetyltransferase 1 (NAT1) is a drug metabolising enzyme that has been associated with increased survival in breast cancer patients [3–5]. NAT1 is differentially expressed in breast cancer subtypes, being highest in those of luminal origin and lowest in basal-like tumours [6]. A study of almost 2000 breast cancer patients found the presence of three genetically distinct populations based on NAT1 expression alone [5]. Overall survival in these populations differed significantly, where the upper quartile survival was 3.2 and 8.4 years for patients in the lowest and highest NAT1 groups, respectively. In those patients treated with chemotherapy, there was a strong positive relationship between tumour NAT1 expression and survival over the first 5 years post-treatment. This association was not seen in patients who did not receive chemotherapy. Almost all patients who survived less than 2 years had tumours with no detectable NAT1 mRNA, suggesting NAT1 deficiency is a strong predictor of poor drug response.

Apart from its role in drug metabolism, NAT1 has been shown to affect cancer cell morphology, growth and proliferation, metastasis and invasion, and cellular bioenergetics [7–10]. However, the mechanism for these effects is not well understood. The loss of NAT1 increased SNAIL expression in a range of breast cancer cells, whereas both N-cadherin and β-catenin were reduced [9]. The metalloproteinase MMP9 was also up regulated in cells deficient in NAT1 [11]. The loss of NAT1 results in changes to mitochondrial function [7, 10]. Mitochondria regulate the intrinsic apoptotic pathway and changes in mitochondrial function can lead to drug resistance [12].

One aspect that has not been addressed is the contradiction between phenotypes revealed through NAT1-deficient cells and microarray studies. The loss of NAT1 results in reductions in growth, proliferation, invasion, and metastasis. However, high tumour NAT1 expression is associated with better patient outcomes and survival. It is a possibility that, while high NAT1-expressing breast cancers present a more aggressive phenotype, they are easier to treat with conventional methods that target cells undergoing rapid proliferation. It has been postulated that this discrepancy could be due to the unknown effect of NAT1 on chemotherapeutic sensitivity [5].

Currently, there are no studies that have systematically investigated the effect of NAT1 on drug sensitivity. Analysis of available microarray data in public databases (GEO Profiles) shows conflicting results. For example, low NAT1 expression is associated with enhanced docetaxel and doxorubicin resistance in PC3 prostate cancer cells and MCF-7 breast cancer cells, respectively [13, 14]. By contrast, HT-29 colorectal adenocarcinoma cells resistant to methotrexate showed elevated NAT1 compared to sensitive cells [15]. Similarly, A2780 epithelial ovarian cancer cells with high NAT1 expression were resistant to platinum compounds [16]. The limited scope of current studies has made it difficult to draw any conclusion on whether a causal relationship exists between tumour NAT1 expression and drug sensitivity. Moreover, these studies provide little mechanistic basis for any NAT-dependent resistance.

The present study was designed to investigate whether NAT1 deficiency seen in a significant proportion of breast tumours affects anti-cancer drug response. Seven different drugs were investigated in four breast cancer cell lines – triple negative MDA-MB-231 cells, estrogen receptor positive T-47D cells, NAT1 deficient MDA-MB-231 cells and NAT1 deficient T47D cells. Mitochondrial-dependent apoptosis was investigated in detail because of previously reported changes in mitochondrial function in NAT1-deficient cells.

### Materials and methods

#### Cell culture

MDA-MB-231 and T-47D cells were purchased from the American Type Culture Collection and maintained in RPMI 1640 medium supplemented with 10% foetal calf serum (HyClone), 2 mM glutamine and 100 units/ml penicillin/streptomycin (Thermo Fisher Scientific) at 37 °C with 5% CO_2_.

#### CRISPR/Cas9 knockout of NAT1

Disruption of the NAT1 gene was achieved through a human NAT1 gene knockout (KO) CRISPR/Cas9 kit (OriGene Technologies, KN221042). This kit contained two gRNA vectors, a donor vector with the green fluorescent protein gene and puromycin selection cassette and a scramble gRNA vector to act as a control. Cells were co-transfected with 0.5 µg each of one gRNA vector and donor vector using Lipofectamine 2000 (Thermo Fisher Scientific) for 4 h. Following transfection, cells were passaged ten times before selection with 0.5–1 µg/ml puromycin. Resistant colonies were selected and screened for NAT1 activity via the previously described method [17]. Gene deletion was also confirmed by Western blot and by PCR of genomic DNA as described elsewhere [18].

#### Re-insertion of NAT1 gene into NAT1 KO cells

NAT1 was rescued in MDA-MB-231 cells by stable transfection of pcDNA3-NAT1 vector. This vector contained the NAT1 gene sequence and a geneticin selection cassette. NAT1 KO cells were transfected with 1 µg of linearised vector using Lipofectamine 2000 (Thermo Fisher Scientific) for 4 h. Following transfection, cells were passaged ten times before selection with 1 mg/ml geneticin (Sigma-Aldrich). Resistant colonies were selected and screened for NAT1 activity [17]. Similar experiments were attempted in T-47D cells, but no viable colonies were obtained.

#### Preparation of drugs

Cisplatin, epirubicin, etoposide, 5-fluorouracil (5-FU), paclitaxel, N-acetylcysteine (NAC) and 2',7'-dichlorodihydrofluorescein diacetate (DCFDA) were purchased from Sigma-Aldrich. Daunorubicin was obtained from the National Cancer Institute (Bethesda, Maryland). Necrosulfonamide, necrostatin and Z-IETD-FMK were purchased from Selleck Chemicals. Each of these compounds were dissolved in dimethyl sulfoxide (DMSO) or 100% ethanol (paclitaxel), with the final solvent concentrations not exceeding 1%. Cisplatin stocks were made fresh to prevent DMSO-induced inactivation. All compounds were further diluted in RPMI 1640 medium.

#### Cell proliferation measurements

The CyQuant NF Cell Proliferation Kit (Thermo Fisher Scientific) was used to assess the response to chemotherapeutics. MDA-MB-231 and T-47D cells were seeded in 96-well plates at 5 × 10^3^ and 2.5 × 10^3^, respectively, in medium containing 0.2 mM glutamine and cultured overnight. Cells were treated with DMSO or increasing concentrations of drug for 72 h. For experiments involving a second compound, cells were treated with increasing concentrations of drug with and without a single concentration of the second compound. Following the removal of the medium, cells were incubated for 30 min at 37 °C with 50 µl of fluorescent dye reagent in Hank’s balanced salt solution, supplemented with 20 mM 4-(2-hydroxyethyl)-1-piperazineethanesulfonic acid (HEPES) and 35 mg/ml NaHCO_3_. Fluorescence was measured at an excitation wavelength of 485 nm and emission wavelength of 520 nm.

#### Annexin V measurements

Cells were seeded in 12-well plates at 1 × 10^5^ cells/well and cultured overnight. Cells were then treated with a single concentration of drug for 72 h, harvested with trypsin, centrifuged at 2000 × g for 2.5 min and resuspended in 200 µl of annexin V binding buffer (Biolegend). A working stock of dye was prepared with a 1:100 dilution of annexin V dye (Biolegend) and a 1:10 dilution of 7-aminoactinomycin D (Biolegend) in annexin V binding buffer and 10 µl of this stock was mixed with 50 µl of the harvested cells for 20 min at room temperature. Samples were made up to a final volume of 200 µl with annexin V binding buffer and fluorescence measured by flow cytometry (Guava Muse Cell Analyser, Luminex). Gating was set using untreated cells. Early and late apoptotic populations were combined to obtain the percent annexin V positive population.

#### Caspase 3/7 activation

Cells were treated and harvested in 12-well plates as described above and resuspended in 200 µl of RPMI 1640 medium. A working stock of dye was prepared with a 1:20 dilution of Caspase 3/7 dye reagent (Biotium) and a 1:10 dilution of 7-aminoactinomycin D. Then, 10 µl of this stock was mixed with 50 µl of the harvested cells and incubated for 30 min at 37 °C. Samples were made up to a final volume of 200 µl with RPMI 1640 medium and fluorescence measured by flow cytometry (Guava Muse Cell Analyser, Luminex). Gating was set using untreated cells. Apoptotic and apoptotic/dead populations were combined to obtain the population of cells with caspase 3/7 activation.

#### Isolation of the cell cytosolic fraction

Cells were trypsinised, centrifuged at 2000 × *g* for 2.5 min and the pelleted cells washed with PBS. After another centrifugation, cells were resuspended in NEH buffer (1.5 M NaCl, 2 mM EDTA and 200 mM HEPES) with 2 mM MgCl_2_ and 2 mM dithiothreitol. The cell membranes were disrupted with 30 µg/ml (MDA-MB-231) or 100 µg/ml (T-47D) digitonin and cellular debris was removed by centrifugation (10,000 × *g* at 4 °C for 10 min). The supernatants containing the cytosolic proteins were collected and boiled with Laemmli’s buffer for 5 min.

#### Immunoblotting

Cells were washed once with PBS and lysed directly in Laemmli’s buffer containing protease inhibitors (Sigma-Aldrich). The lysates were then collected and boiled for 5 min. Lysates were electrophoresed at 200 V for 30 to 45 min on 12% SDS-acrylamide gels, transferred to a nitrocellulose membrane at 350 mA for 1 h and blocked in 5% skim milk/ PBS with 0.05% Tween-20 (PBST) overnight at 4 °C. Membranes were then washed 3 × 15 min with PBST and rocked overnight at 4 °C with primary antibodies, obtained from Cell Signaling Technology (cytochrome C: 11940S, caspase 8: 9746 T, caspase 9: 9502 T, tubulin: 3873 T and β-actin: 3700 T) or Abcam (NAT1: ab109114). After further washing, membranes were incubated with horseradish peroxidase-conjugated secondary antibodies (Jackson Laboratories, anti-rabbit: 111–035-003 and anti-mouse: 115–035-003) for 1 h at room temperature. Proteins were detected using Westar ETA C 2.0 and Westar Supernova enhanced chemiluminescence reagent (Cyanagen). Protein expression from immunoblots was quantified by densitometry using Image J software.

#### Effect of caspase 8 inhibitor Z-IETD-FMK

The effectiveness of the caspase 8 inhibitor at preventing caspase 8 cleavage was tested in the MDA-MB-231 parental cell line. Cells were treated with concentrations of Z-IETD-FMK up to 30 µM for 72 h. Protein detection was completed by Western blot as described. Cytotoxicity and caspase 3/7 activation in response to cisplatin in the presence and absence of 30 µM Z-IETD-FMK was measured as described above.

#### Statistical analysis

All data are expressed as mean ± SD. Parameters for each of the concentration- response curves were estimated by non-linear regression analysis in GraphPad Prism 9. A four-parameter logistic model was fitted to the data and convergence was confirmed by commencing the regression from at least 2 independent sets of initial parameter values. The estimated parameters were the IC_50_, the Hill coefficient and the terminal plateau. The fourth parameter (maximum proliferation) was fixed at 100% after normalisation of the data to untreated cells.

The area under the concentration response curves was calculated by the trapezoid rule and 95% confidence intervals were calculated by the method of Gagnon and Peterson [19]. Resistance was calculated as the fold change in area under the curve (AUC) in the NAT1 KO cells compared to the parental cells. Significant differences were determined when the 95% confidence intervals did not overlap. Significance differences for all other data were determined using Student’s t test, one-way or two-way ANOVA with Tukey’s correction for multiple comparisons. Significance was assumed at *p* < 0.05.

### Results

#### Deletion of NAT1 in MDA-MB-231 and T-47D cells

**Fig. 1:**
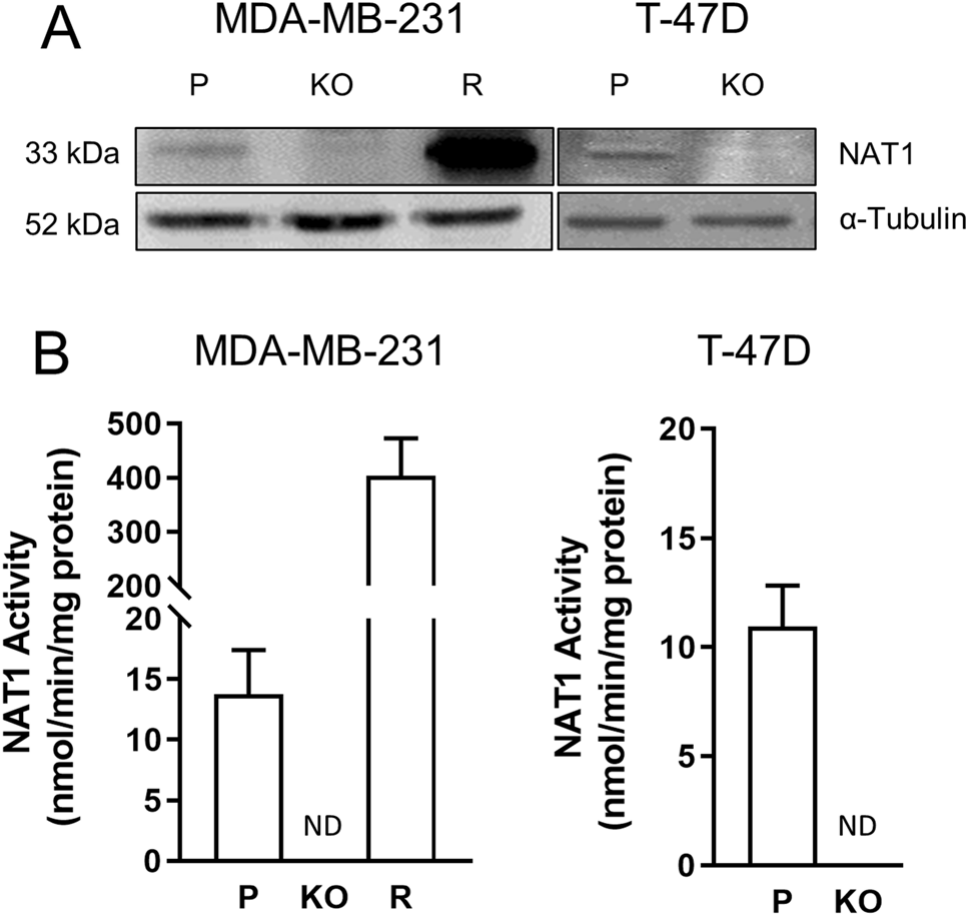
CRISPR/Cas9 generated NAT1 knockout breast cancer cell lines. **A** NAT1 protein expression in the five cell lines used in this study. **B** NAT1 activity in each of the cell lines used. Data are mean ± SD (*n* = 3). ND indicates that activity was not detected. *P* = parental, *KO* = NAT1 knockout and *R* = NAT1 rescue

NAT1 was disrupted in the triple-negative MDA-MB-231 cells and the estrogen receptor positive T-47D cells using CRISPR/Cas9 gene editing. The absence of NAT1 expression in each cell line was confirmed by Western blot for NAT1 protein where no protein was detected (Fig. 1A), the absence of NAT1 enzymatic activity (Fig. 1B) and PCR of genomic DNA as described elsewhere [18]. To rescue the effects of NAT1 gene deletion, NAT1 was re-inserted into the NAT1 KO cells by stable transfection of a construct containing the NAT1 gene under the control of the CMV promoter. This resulted in a cell line with activity approximately 40 times that in the parental cells (Fig. 1B). These cells were used to determine whether changes in drug sensitivity could be reversed. NAT1 was rescued in the MDA-MB-231 cells but, despite several attempts, no viable colonies were obtained in the T-47D cells. Previous studies have reported that T-47D cells are resistant to geneticin selection, which may explain the lack of colony formation [20]

#### Drug response in NAT1 deficient cells

**Fig. 2:**
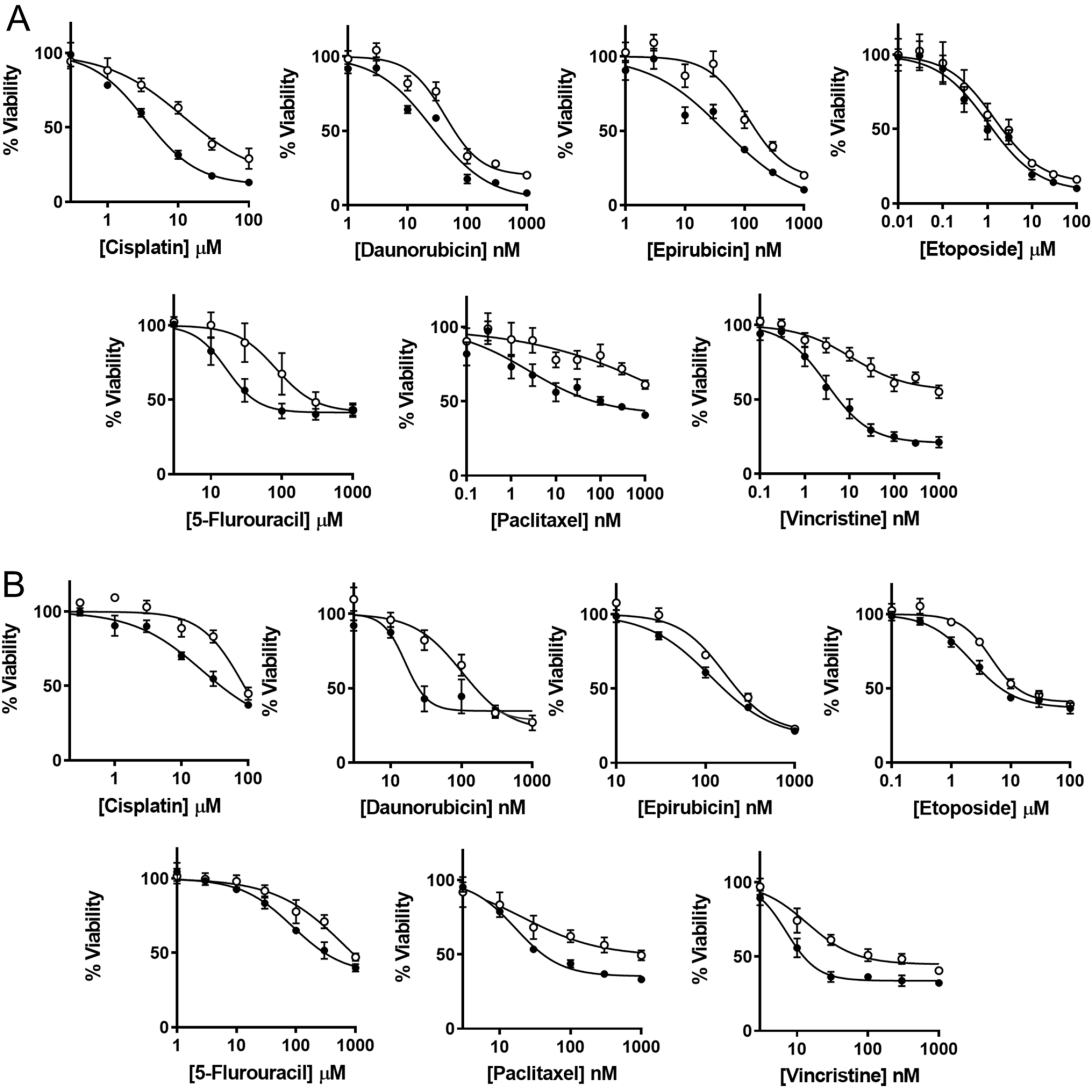
Effect of NAT1 deletion on cytotoxicity of chemotherapeutic agents in breast cancer cell MDA-MB-231 (**A**) and T-47D (**B**) cells. Parent (closed symbols) and NAT1 KO (open symbols) cells were treated with a range of concentrations of seven chemotherapeutic agents over 72 h. Cytotoxicity was assessed with CyQuant Cell Proliferation kit. Data are mean ± SD (*n* = 4), normalised to untreated cells

**Fig. 3:**
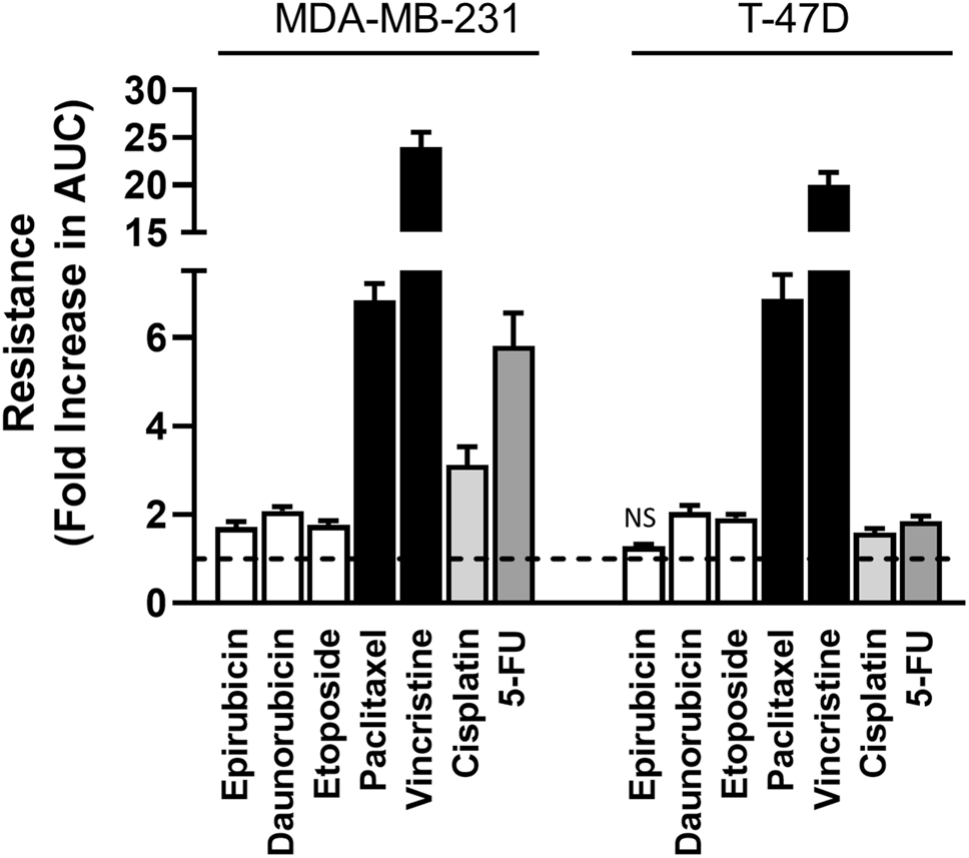
Resistance of NAT1-deficient cells to the cytotoxicity of anticancer drug. Resistance was quantified as the fold change in the AUC in the KO cells to that in the parent cells. Data are mean ± SD (*n* = 4). Significant change in AUC between parent and NAT1 KO was seen for all treatments except epirubicin in the T-47D cells (NS = not significant). Data are grouped according to drug class. White = topoisomerase inhibitors; black = microtubule inhibitors; light grey = DNA cross-linker; dark grey = anti-metabolite

Cytotoxicity to seven different anti-cancer drugs was assessed over 72 h in all 4 cell lines (Fig. 2A and B). The drugs used were cisplatin, daunorubicin, epirubicin, etoposide, 5-FU, paclitaxel and vincristine. They were selected to represent different drug classes that are used, or are under investigation, for the treatment of breast cancer. The deletion of NAT1 shifted the dose–response curves to the right in both cell lines, indicating a decrease in potency. The parameters for each dose–response curve are provided in Supplementary Tables 1 and 2. For most drugs, both the IC_50_ and the terminal plateau changed in the NAT1 KO lines indicating a decrease in potency as well as efficacy. To account for these changes, the area under the curve (AUC) was used to compare the different drugs as this measurement is independent of the shape of the curve [21]. Resistance was quantified as the increase in the AUC compared to the parental cells and varied between drug classes (Fig. 3). There was a two-fold increase in AUC for the topoisomerase inhibitors (epirubicin, daunorubicin, etoposide) in each cell line following NAT1 deletion, except for epiriubicin in the T-47D cells. By contrast, resistance to the microtubulin inhibitors increased sevenfold (paclitaxel) and 20-fold (vincristine). Cisplatin was 2 to fourfold less effective in the NAT1 KO cells and the anti-metabolite 5-FU was 2 to sixfold less effective.

#### NAT1 rescue and drug response

**Fig. 4:**
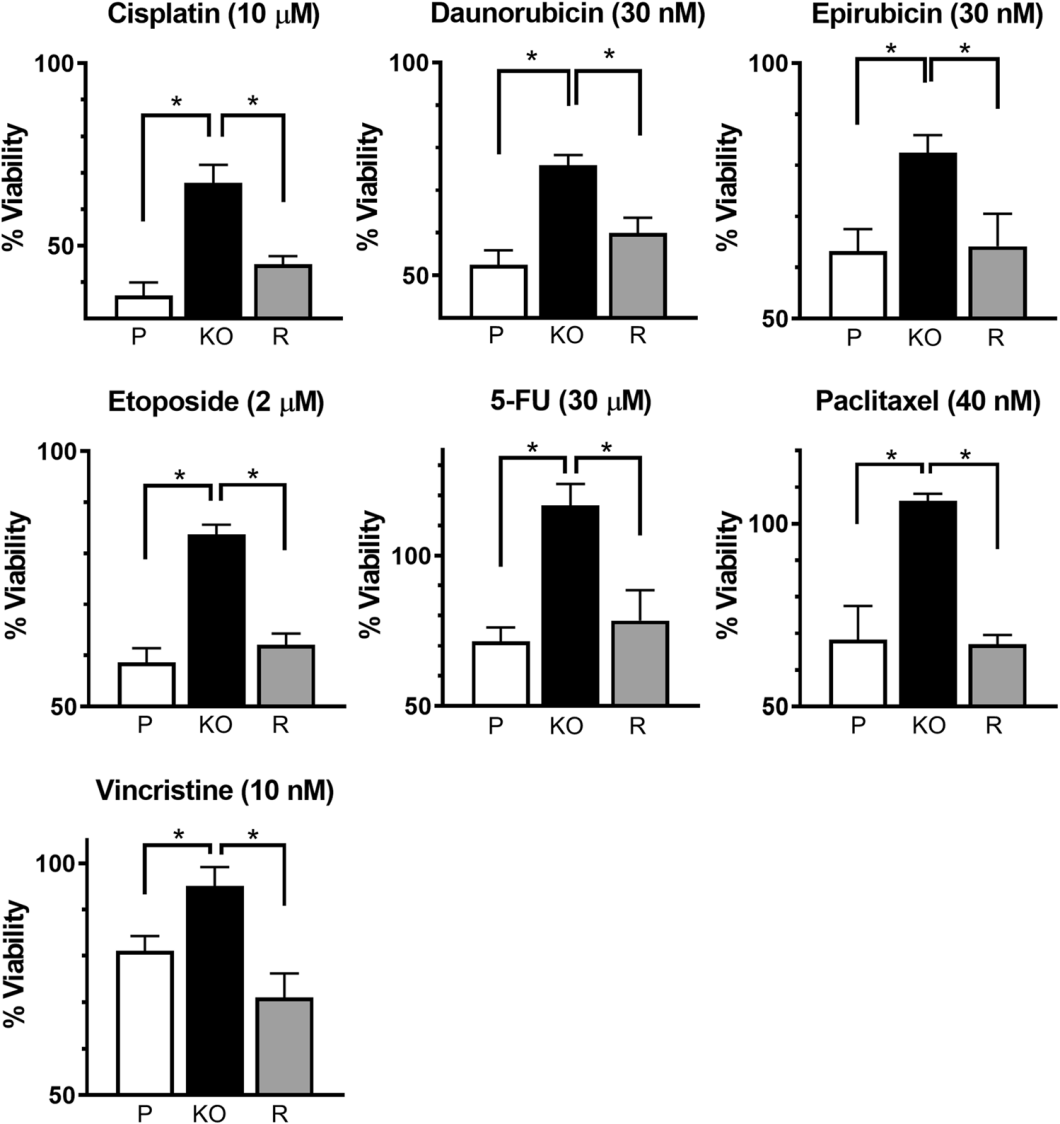
NAT1 re-insertion rescues chemotherapeutic sensitivity in MDA-MB-231 cells. MDA-MB-231 parent (P), NAT1 KO (KO) and NAT1 rescue (R) were treated with a chemotherapeutic agent for 72 h at the concentration specified for each graph. Data are means ± SD (*n* = 4), normalised to untreated cells (100%). Significance was determined by one-way ANOVA with Tukey’s correction for multiple comparisons and is indicated by an asterisk (*)

NAT1 was rescued in the MDA-MB-231 KO cells to determine whether the increased drug resistance was a direct effect of NAT1 deletion, or a result of the selection and cloning procedure used to develop the NAT1 deleted line. The rescued cells showed the same cell death as the parental cells for all seven drugs indicating reversal of drug resistance (Fig. 4). It is noteworthy that the rescued cells expressed NAT1 approximately 40-fold higher than the parental cells (Fig. 1), suggesting that increasing NAT1 activity beyond that in the parental cells does not further sensitise the cells.

#### Inhibition of apoptosis following NAT1 deletion

**Fig. 5:**
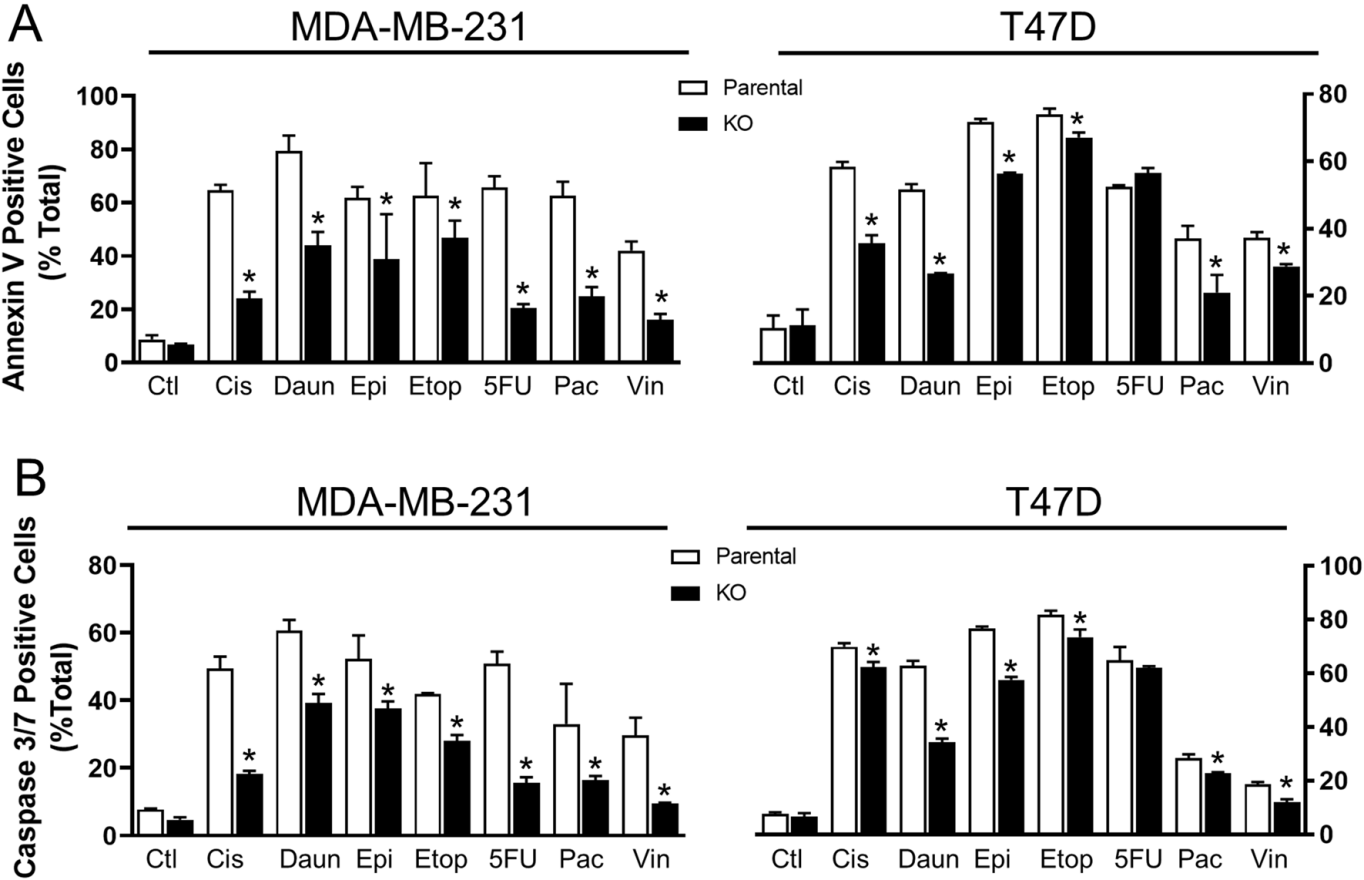
Apoptosis in breast cancer cells following treatment with chemotherapeutic agents. Parent (open bars) and NAT1 KO (closed bars) cells were treated with each drug for 72 h following which annexin V (**A**) or caspase 3/7 activity (**B**) was quantified. Drug concentrations for MDA-MB-231 cells, see Fig. 4. For T-47D cells, cisplatin = 20 µM, daunorubicin = 70 nM, epirubicin = 180 nM, etoposide = 15 µM, 5-FU = 500 µM, paclitaxel = 100 nM, vincristine = 10 nM. Data are means ± SD, (*n* = 3). Significance between parent and NAT1 KO cells was determined by two-way ANOVA with Tukey’s correction for multiple comparisons and is indicated by an asterisk (*)

Apoptosis was assessed by measuring phosphatidylserine externalisation using annexin V binding in each cell line following drug treatment (Fig. 5A). Except for 5-FU in the T-47D cells, annexin V binding was significantly less in the NAT1 KO cells compared to the parental cells, although the differences were smaller than the changes seen in cell viability. Phosphatidylserine externalisation results, in part, from caspase-dependent inactivation of flippases, although other proteases can also cleave this protein. To specifically measure apoptosis, activation of the executioner caspases 3 and 7 were quantified (Fig. 5B). All drugs enhanced caspase 3/7 activity in the parental cells. Similar to the annexin V binding results, NAT1 deletion significantly decreased caspase 3/7 activity, except for 5-FU in the T-47D cells. These results suggest that NAT1 deficiency inhibits drug-induced apoptosis.

#### NAT1 deficiency and activation of caspase 8 and 9

**Fig. 6:**
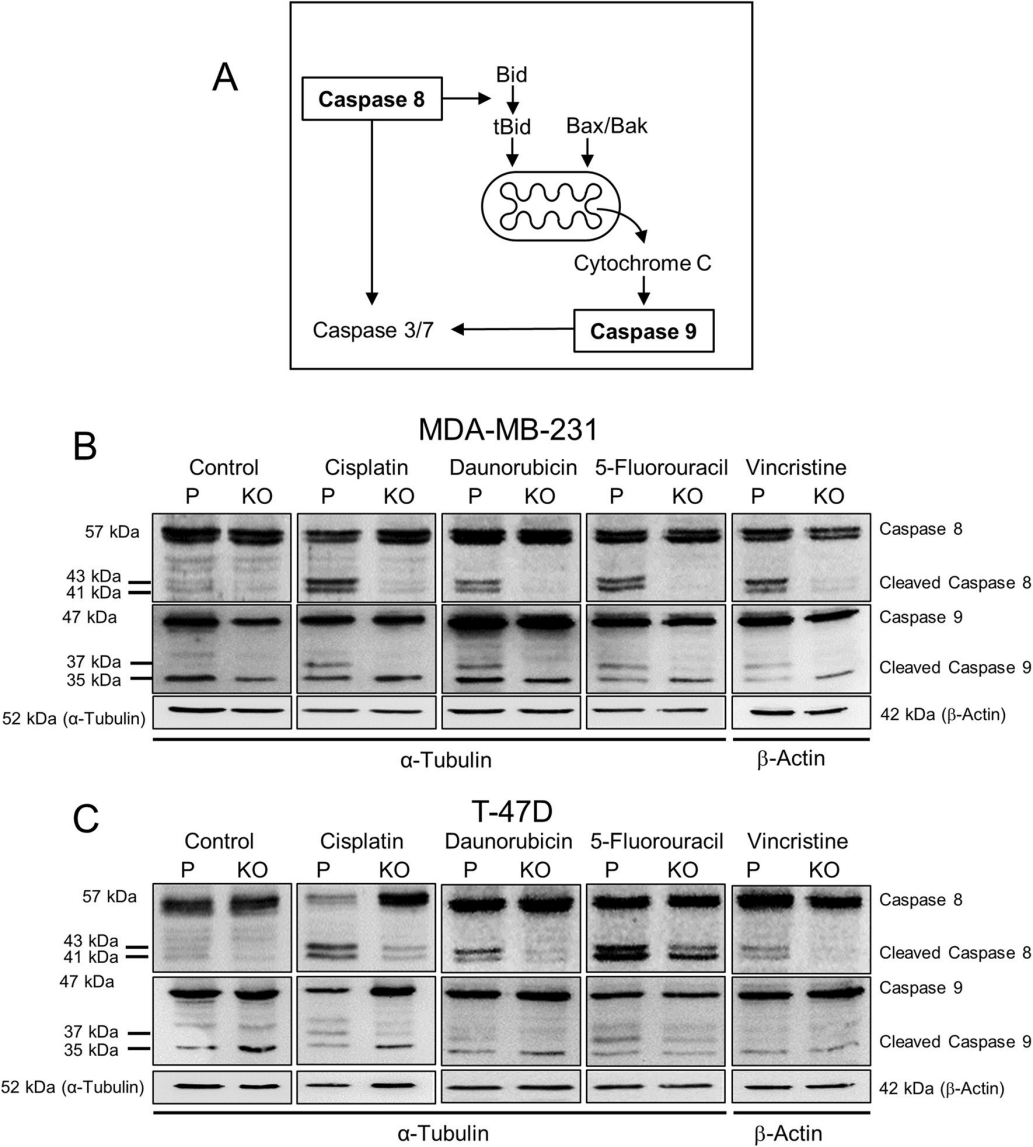
Effect of NAT1 deletion on drug-induced caspase 8 and 9 activation. **A** Schematic showing the release of cytochrome C from mitochondria and activation of the executioner caspase 3/7 by caspase 8 (extrinsic apoptosis pathway) and caspase 9 (intrinsic apoptosis pathway). Caspase 8 can also activates Bid, which is upstream of caspase 9. Adapted from Shalini et al. [38]. MDA-MB-231 (**B**) and T-47D (**C**) cells were treated with a single concentration of each of four chemotherapeutic agents for 72 h. For the concentration of each drug, refer to Fig. 5. *P* = parental, KO = NAT1 knockout. Expression of pro- and cleaved forms of both caspase 8 and 9 was measured by SDS-PAGE/Western blot. Blots are representative of two independent experiments. β-actin was used as the loading control for vincristine due to its effects on α-tubulin expression

Both the extrinsic and the intrinsic apoptotic pathways activate caspase 3/7, the first via caspase 8 and the second via caspase 9 (Fig. 6A). The effect of NAT1 deficiency on these two caspases was investigated in the parental and NAT1-deleted cells (Fig. 6B and C). NAT1 did not affect pro-caspase 8 in any of the cells and drug-induced activation was evident in both parental cell lines by cleavage of the 57 kDa procaspase 8 to the active 41 and 43 kDa caspase 8. By contrast, cleavage was minimal in the NAT1-deficient cells for all drugs tested.

Caspase 9 was also activated in the parental cell lines following drug treatment, indicated by the appearance of the 37 kDa cleaved form. This activation was attenuated in the cells following NAT1 deletion. These results show that loss of NAT1 inhibits caspase 8 and 9 activation to downregulate both major apoptotic pathways.

#### NAT1 deficiency and drug-induced cytochrome C release

**Fig. 7:**
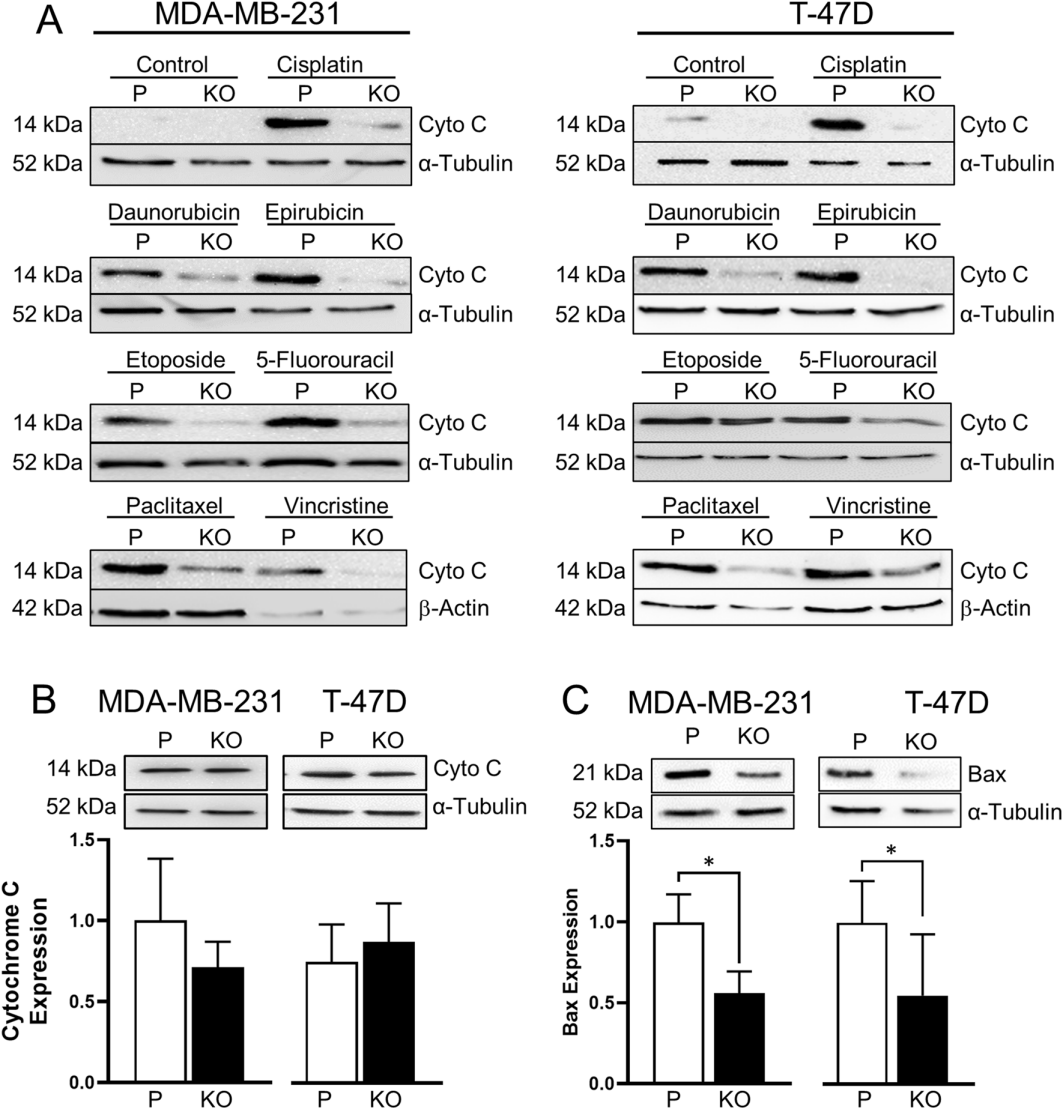
Effect of NAT1 deletion on drug-induced cytochrome C release. A. MDA-MB-231 and T-47D cells were treated with each drug for 72 h. For the concentration of each drug, refer to Fig. 5. *P* = parental, KO = NAT1 knockout. Cytosols were isolated and cytochrome C measured by Western blot. Blots are representative of at least two independent experiments. α-Tubulin was used as the loading control, except for paclitaxel and vincristine experiments where β-actin was used due to their effects on α-tubulin expression. B. Total cytochrome C, measured by Western blotting, in each cell line was not different (*p* > 0.05, *n* = 3). C. Bax expression in whole cell extracts. Asterisk indicates significant difference (*p* < 0.05, *n* = 3)

Activation of caspase 9 requires cytochrome C release from mitochondria (Fig. 6A). Drug-induced increase in cytosolic cytochrome C was seen in both the MDA-MB-231 and T-47D parental cells (Fig. 7A). However, for all drugs tested, this was reduced following NAT1 deletion. The lower cytosolic cytochrome C levels were not a result of less total cytochrome C in the NAT1 KO cells, as shown by Western blot of whole cell extracts (Fig. 7B).

During intrinsic apoptosis, Bax oligomerises with Bak to form a pore in the outer mitochondrial membrane to initiate cytochrome C release (Fig. 6A). To determine whether Bax expression was affected by NAT1 deficiency, Bax protein was quantified in whole cell extracts (Fig. 7C). In both the MDA-MB-231 and T-47D cells, Bax decreased following NAT1 deletion. These results suggest that a lower intracellular level of Bax may be responsible for the decrease in cytochrome C release following drug treatment, which in turn inhibits caspase 9 activity and caspase 3/7 activation.

#### Role of Caspase 8 in parental and NAT1-deficient cells

**Fig. 8:**
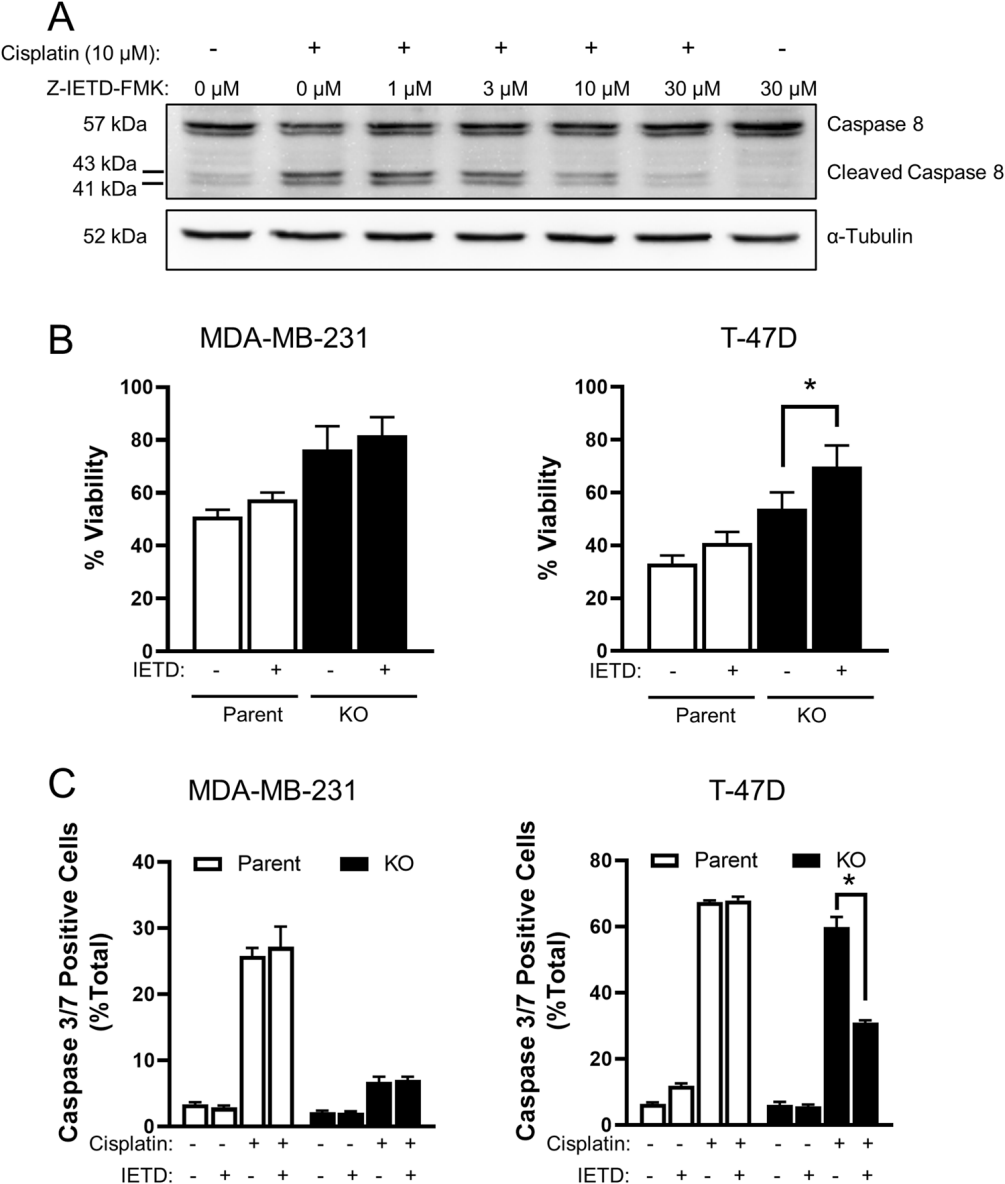
Effect of caspase 8 inhibitor, Z-IETD-FMK, on response to cisplatin. **A** MDA-MB-231 parent cells were treated with cisplatin (10 µM) and increasing concentrations of Z-IETD-FMK for 72 h. Expression of pro- and cleaved caspase 8 was measured by SDS-PAGE/Western blot. α-Tubulin was used as the loading control. **B** MDA-MB-231 (left) and T-47D (right) parent and NAT1 KO cells were treated with 10 µM (MDA-MB-231) and 20 µM (T47D) cisplatin with and without 30 µM Z-IETD-FMK for 72 h. Data are mean ± SD (*n* = 4), normalised to untreated cells. **C** MDA-MB-231 (left) and T-47D (right) parent (open bars) and NAT1 KO (closed bars) cells were treated with a single concentration of cisplatin with and without the Z-IETD-FMK for 72 h and stained for caspase 3/7 activity. Fluorescence was measured with a Muse Cell Analyser. Data are mean ± SD (*n* = 3). Significance was determined by two-way ANOVA with Tukey’s correction for multiple comparisons, with significance set at 0.05, indicated by an asterisk (*)

The mechanism of drug resistance following NAT1 deletion was further investigated using cisplatin as a model drug. To assess the role of caspase 8 in cell death, both MDA-MB-231 and T-47D cells were treated with cisplatin for 72 h in the absence and presence of increasing concentrations of Z-IETD-FMK, an inhibitor of caspase 8 activation. Inhibition was effective at 30 μM Z-IETD-FMK (Fig. 8A), which was then used for further experiments. The inhibitor did not affect cisplatin-induced toxicity in either the MDA-MB-231 or T-47D parental cells (Fig, 8B) suggesting caspase 8 did not contribute to cell death. Similarly, it had no effect in the MDA-MB-231 NAT1 KO cells. However, Z-IETD-FMK significantly increased cell viability in the T-47D NAT1 KO cells, although there was not complete protection. To see what effects Z-IETD-FMK had down-stream of caspase 8, caspase 3/7 activation was also quantified. As seen with total cell viability, the inhibitor partially reversed caspase 3/7 activation in the T-47D NAT1 KO cells. These results suggest that the extrinsic apoptotic pathway has little role in cisplatin toxicity in both parental cell lines, and that the activation of caspase 3/7 is primarily caspase 9-dependent. Nevertheless, it may contribute, in part, to drug toxicity following NAT1 deletion, at least in the T-47D cells. The increased resistance to cisplatin seen in the absence of NAT1 is a result of the reduced caspase 9 activity.

#### Effect of necroptosis inhibitors on cisplatin toxicity

**Fig. 9:**
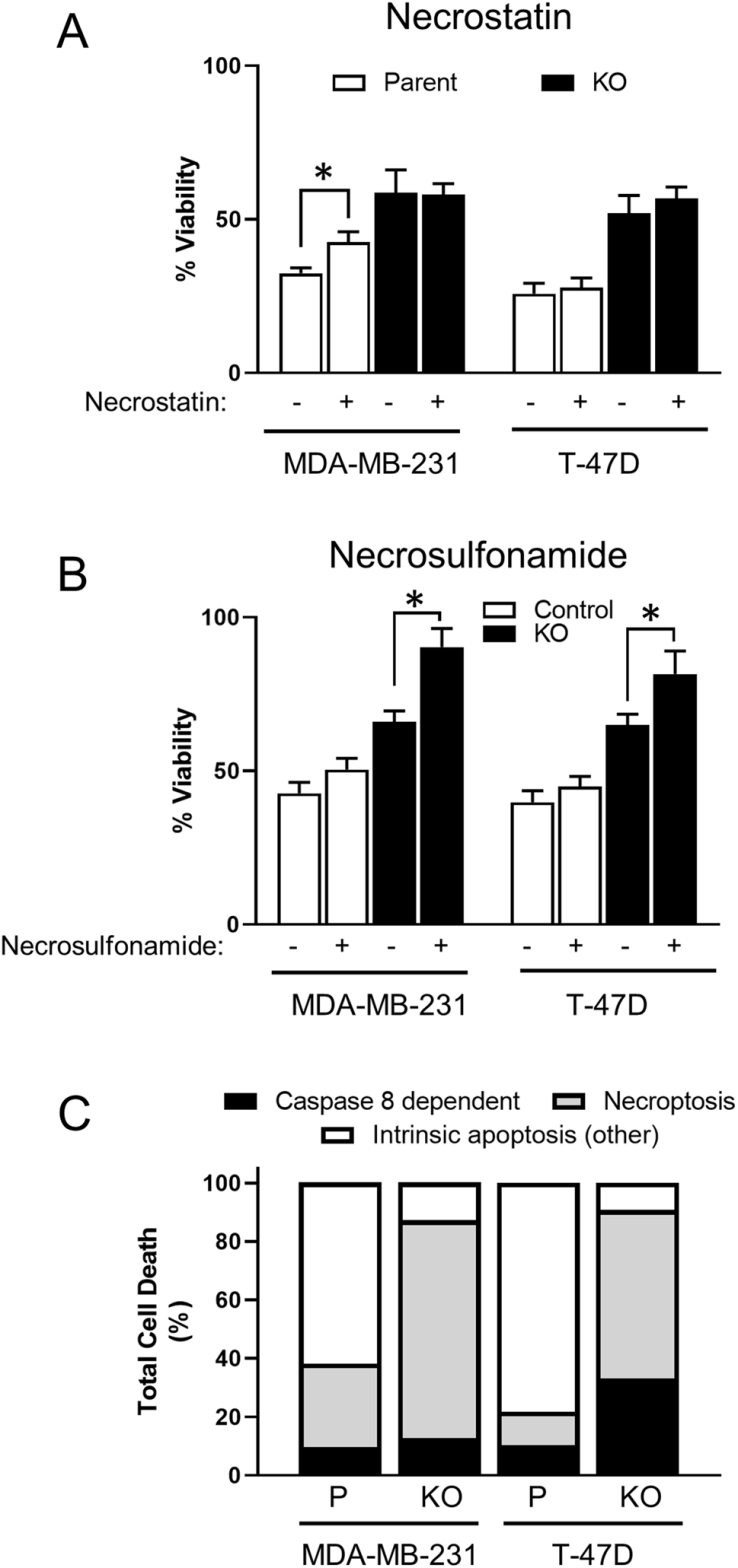
Effect of necroptosis inhibitors on cisplatin cytotoxicity. MDA-MB-231 and T-47D parent (open bars) and NAT1 KO (closed bars) cells were treated with cisplatin with and without necrostatin (50 µM) **(A)** or necrosulfonamide (3 µM) **(B)** for 72 h. Cytotoxicity was assessed with the CyQuant Cell Proliferation kit. Data are means ± SD (*n* = 4), normalised to untreated cells. **C** Proportion of cell death in each cell line attributed to caspase 8 (closed bar), necroptosis (grey bar) and intrinsic apoptosis (open bar). Caspase 8 dependent cell death was calculated from data in Fig. 8, necroptosis was calculated from data in Fig. 9 A and B, and intrinsic apoptosis was calculated as the remaining proportion of cell death, which may include other less common pathways such as ferroptosis or pyroptosis

In addition to triggering the apoptotic pathways, cytotoxic drugs can induce cell death by necroptosis [22] especially in cells that evade apoptosis [23]. To determine whether NAT1 deletion altered the necroptotic response to cisplatin, the effects of necrostatin (RIPK1 inhibitor) and necrosulfonamide (RIPK3 inhibitor) were investigated. Necrostatin partially protected the MDA-MB-231 parental cells treated for 72 h with 10 µM cisplatin, increasing viability from 32 to 42% (Fig. 9A). It had no effect on MDA-MB-231 KO cells or the T-47D cells. These results suggest that RIPK1 has little or no role in cisplatin cytotoxicity, at least in these cells. By contrast, necrosulfonamide did not affect toxicity in either parental line, but it significantly protected the two NAT1 deleted lines (Fig. 9B). In the MDA-MB-231 NAT1 KO cells, viability increased from 65 to 90% and, in the T-47D KO cells, it increased from 65 to 82%.

In Fig. 9C, the contributions of caspase 8-dependent, necroptosis and other possible pathways, which includes intrinsic apoptosis, to cisplatin-induced cell death have been summarised from the data shown on Figs. 8B, 9A and B. In both parental cell lines, the non-necroptosis or caspase 8-independent pathways predominated accounting for 62% and 78% of cell death in the MDA-MB-231 and T-47D cells, respectively. Caspase 8-dependent cell death accounted for approximately 10%. By contrast, in the NAT1 KO cells, necroptosis was the major pathway of cell death accounting for 75% in the MDA-MB-231 cells and 58% in the T-47D cells. Taken together, these results show that both cancer cell lines switch between apoptosis and necroptosis when NAT1 expression is absent.

##### Discussion

Approximately, 30% of tumours from breast cancer patients have undetectable levels of NAT1 mRNA. These patients have the lowest overall survival and the poorest response to chemotherapy [5]. The results of the present study show that NAT1 deficiency in breast cancer cells increased resistance to a range of chemotherapeutic agents regardless of their mechanism of action. Some, such as the topoisomerase inhibitors showed modest resistance whereas other such as the microtubulin inhibitors showed much greater resistance. The loss of drug sensitivity was rescued for all seven drugs when NAT1 was re-introduced into the knockout cells. This strongly argues that NAT1 deficiency is causative of drug resistance in this study. Interestingly, over-expression of NAT1 did not further sensitise the cells.

**Fig. 10:**
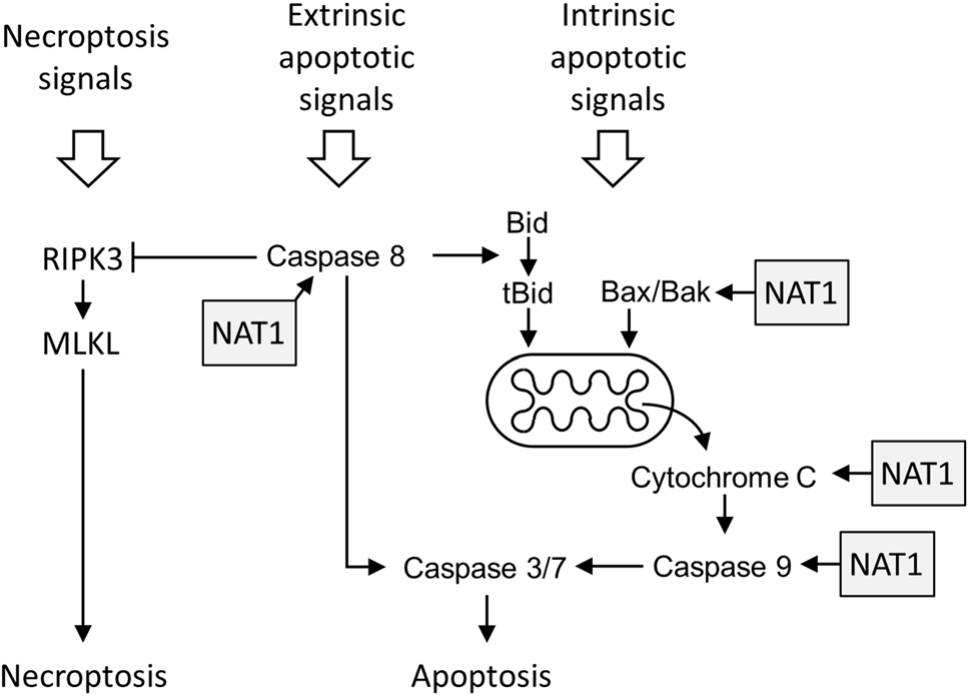
Schematic showing the intersection of the apoptotic and necroptotic pathways in cell death and the location of individual steps that are proposed to be affected by NAT1

The loss of NAT1 resulted in down-regulation of caspase 9 activation, the major initiator caspase in the intrinsic apoptotic pathway. This can be explained by the attenuated release of cytochrome C in the NAT1 deficient cells. There have been several reports that implicate NAT1 in mitochondrial function. Wang et al. showed a decrease in pyruvate dehydrogenase activity [10] while Carlisle et al. reported changes in oxidative phosphorylation [7] in mitochondria from NAT1 knockout cells. The attenuation of cytochrome C release in response to cytotoxic drug treatment further suggests fundamental mitochondrial changes in cells with little or no NAT1. In this study, there was a decrease in Bax expression in NAT1 deficiency. Bax oligomerises with Bak to form pores in the outer mitochondrial membrane through which cytochrome C is released [24]. However, depletion of intracellular BAX alone is usually insufficient to prevent cytochrome C release [25]. Further work is needed to quantify other Bcl-2 family proteins in NAT1 deficiency. Taken together, the results suggest that NAT1 is necessary for optimum cell death by drugs that activate the intrinsic apoptosis pathway, but further work is required to identify its mechanism more precisely. The results also imply that the loss of NAT1 in patient tumours may contribute to drug resistance. Along with the loss of caspase 9 activation, caspase 8 was also downregulated in NAT1 depleted cells. Both proteases initiate caspase 3/7 activation and apoptosis (Fig. 10). Consequently, drug-induced cell death in NAT1 depleted cells is unlikely to result primarily from the intrinsic or extrinsic apoptotic pathways.

Cisplatin is a DNA binding drug that induces cell death primarily via intrinsic apoptosis [26]. In patients with triple negative breast cancers, cisplatin significantly improves tumour response [27]. It also demonstrates efficacy in patients with HER2 positive tumours [28] or BRCA1-positive tumours [29]. To further investigate possible underlying mechanism(s) of drug resistance in NAT1 deficiency, cisplatin was studied in greater detail. Deletion of NAT1 shifted the cisplatin dose–response curve to the right in both cell lines (Fig. 2) with the IC_50_ increasing fourfold (Supplementary tables 1 and 2). Like the other drugs used, cytochrome C release, caspase 9 activation and caspase 8 activation were diminished in NAT1 knockout cells when treated with cisplatin. However, in both the MDA-MB-231 and T-47D parental cells, caspase 8 activity was not required for caspase 3/7 activation or cell death (Fig. 8). Following NAT1 deletion, the T-47D cells partially switched to caspase 8-dependent cell death. However, this was not seen in the MDA-MB-231 cells. This further demonstrates switching from apoptosis to another pathway in the absence of NAT1. When necroptosis was evaluated, the RIPK3 inhibitor necrosulfonamide almost completely inhibited cisplatin-induced cell death in the NAT1 deficient cells but had no effect in the parental cells. We concluded from these results that cells lacking NAT1 switch from the classical apoptosis pathways to alternative cell death pathways when treated with cytotoxic drugs, and this may contribute to the observed drug resistance. This switching is summarised in Fig. 9C which shows that parental cells die primarily by intrinsic apoptosis whereas the NAT1 deficient cells die primarily by necroptosis when treated with cisplatin.

Many reports have demonstrated flexibility between the different cell death pathways [30]. For example, caspase 8 initiates extrinsic apoptosis but can also activate mitochondrial-dependent apoptosis through the cleavage of Bid (Fig. 10) [31]. Apoptosis is a high energy requiring pathway because ATP is needed for caspase activation, chromatin condensation and membrane bleb formation. Under stress, cells can switch to necroptosis when intracellular ATP levels are low [32]. NAT1 modifies oxidative phosphorylation [7, 10], which may affect intracellular ATP. Moreover, NAT1 activity is allosterically regulated by ATP, which competes with the acyl donor acetyl coenzyme A [5]. This relationship between NAT1, ATP and cell death is an attractive hypothesis but requires further studies to quantify the effects of NAT1 expression on intracellular ATP, especially in the presence of cytotoxins such as the drugs used in the present study.

Although caspase 8 was not required for cell death, it may have a role in switching from apoptosis to necrosis when NAT1 is deficient. Caspase 8 suppresses necroptosis by inactivating RIPK3 (Fig. 10) preventing activation of downstream MLKL [33, 34]. Caspase 8 was downregulated in NAT1 deficient cells, which may allow activation of necroptosis. In head and neck squamous cell carcinoma, inhibition of caspase 8 sensitised cells to necroptosis when co-treated with birinapant, a SMAC mimetic [35]. This presents a possible therapeutic intervention for patients with low or absent NAT1 in their tumours. Birinapant has already shown efficacy in combination with platinum-based drugs in ovarian cancers [36] and with gemcitabine in triple-negative breast cancers [37].

In summary, the current study has shown that loss of NAT1 in breast cancer cells is likely to enhance drug resistance by inhibiting intrinsic apoptosis, possibly leading to the activation of necroptosis as the major cell death pathway. The class-independent effect of NAT1 deficiency may explain the poor response to chemotherapy seen in those patients with low tumour NAT1 expression. The results provide a framework for further studies on the role of NAT1 in drug-induced cell death.

## Supplementary Information

Below is the link to the electronic supplementary material.

Supplementary file1 (PDF 99 KB)

## Data Availability

All datasets are available from the corresponding author upon reasonable request.

## Abbreviations

NAT1: Arylamine *N*-acetyltransferase 1
AUC: Area under the curves
DMSO: Dimethyl sulfoxide
HEPES: 4-(2-Hydroxyethyl)-1-piperazineethanesulfonic acid
