# Supplementary material for: Arylamine N-acetyltransferase 1 deficiency inhibits drug-induced cell death in breast cancer cells: switch from cytochrome C-dependent apoptosis to necroptosis
Source: Breast Cancer Res Treat. 2022 Aug 2;195(3):223–36. doi: 10.1007/s10549-022-06668-3 (PMC9464750; doi:10.1007/s10549-022-06668-3)
Supplement: Supplementary file 1 — Supplementary file1 (PDF 99 KB) [file 10549_2022_6668_MOESM1_ESM.pdf]

Supplementary data

The loss of arylamine N-acetyltransferase NAT1 expression in breast cancer enhances drug resistance

Courtney E. McAleese, Neville J Butcher and Rodney F. Minchin

Supplementary Table 1: Parameters of the dose-response curves in MDA-MB-231 cells

**Drug**	**IC_{50}**		**Hill coefficient**		**Terminal Plateau**	
	Parent	NAT1 KO	Parent	NAT1 KO	Parent	NAT1 KO
Cisplatin (µM)	3.4 ± 0.3	11.7 ± 4.1	-1.16 ± 0.1	-0.81 ± 0.1	11.4 ± 2.5	14.9 ± 8.8
Daunorubicin (nM)	26.0 ± 3.8	42.7 ± 7.3	-0.92 ± 0.1	-1.44 ± 0.3	4.0 ± 3.6	20.0 ± 4.3
Epirubicin (nM)	45.5 ± 16.1	115 ± 23.6	-0.69 ± 0.1	-1.32 ± 0.3	0.7 ± 8.5	17.3 ± 6.6
Etoposide (µM)	1.03 ± 0.2	1.45 ± 0.4	-0.77 ± 0.1	-0.83 ± 0.1	8.1 ± 3.7	13.8 ± 4.9
5-FU (µM)	16.5 ± 1.5	83.0 ± 14.7	-1.94 ± 0.3	1.56 ± 0.34	41.4 ± 1.6	41.8 ± 3.9
Paclitaxel (nM)	2.5 ± 1.1	>1000[1]	-0.52 ± 0.1	-0.3 ± 0.1	41.2 ± 4.5	4.3 ± 15
Vincristine (nM)	3.1 ± 0.4	11.4 ± 3.8	-0.90 ± 0.1	-0.71 ± 0.1	20.8 ± 1.7	56.1 ± 3.1

[1] *estimated values*

Supplementary Table 2: Parameters of the dose-response curves in T-47D cells

Drug	IC_{50}		Hill coefficient		Terminal Plateau	
	Parent	NAT1 KO	Parent	NAT1 KO	Parent	NAT1 KO
Cisplatin (µM)	20.2 ± 6.8	87.0 ± 6.8	-0.85 ± 0.1	-1.36 ± 0.7	21.20± 8.8	0
Daunorubicin (nM)	16.0 ± 1.9	102 ± 24	-2.77 ± 0.5	-1.28 ± 0.3	34.7 ± 2.4	21.4 ± 1.9
Epirubicin (nM)	113 ± 10	165 ± 22	-1.24 ± 0.1	-1.7 ± 0.3	16.8 ± 2.8	20.4 ± 4.9
Etoposide (µM)	2.2 ± 0.2	4.7 ± 0.5	-1.19 ± 0.1	-1.69 ± 0.2	36.9 ± 1.5	40.9 ± 1.9
5-FU (µM)	87.2 ± 18	719 ± 96	-1.0 ± 0.1	-0.7 ± 0.2	35.8 ± 4.2	-
Paclitaxel (nM)	16.7 ± 1.2	22.1 ± 10.5	-1.37 ± 0.1	-0.81 ± 0.2	35.2 ± 1.2	48.9 ± 5.8
Vincristine (nM)	6.9 ± 0.5	13.9 ± 2.3	-1.97 ± 0.2	-1.28 ± 0.2	33.7 ± 1.4	44.7 ± 2.4
